# Association of Socioeconomic Characteristics With Receipt of Pediatric Cochlear Implantations in California

**DOI:** 10.1001/jamanetworkopen.2021.43132

**Published:** 2022-01-14

**Authors:** Rance J. T. Fujiwara, Gail Ishiyama, Akira Ishiyama

**Affiliations:** 1 Department of Head and Neck Surgery, David Geffen School of Medicine at University of California, Los Angeles; 2 Department of Neurology, David Geffen School of Medicine at University of California, Los Angeles

## Abstract

**Importance:**

Earlier cochlear implantation among children with bilateral severe to profound sensorineural hearing loss is associated with improved language outcomes. More work is necessary to identify patients at risk for delayed cochlear implantation and understand targets for interventions to improve cochlear implantation rates among children.

**Objective:**

To describe the demographics among children receiving cochlear implantations and variability in implantation rates in California and to investigate sociodemographic and parental factors associated with early pediatric cochlear implantation.

**Design, Setting, and Participants:**

This retrospective cross-sectional study was conducted using data from the Healthcare Cost and Utilization Project California State Ambulatory Surgery Database in calendar year 2018. Included patients were children aged 9 years old or younger undergoing cochlear implantation. Sociodemographic factors, location of treatment, and parental factors were collected. Data were analyzed from March through August 2021.

**Main Outcomes and Measures:**

Binary logistic regression was performed to investigate sociodemographic factors associated with early cochlear implantation (ie, before age 2 years). Geographic variability in pediatric cochlear implantation across hospital referral regions in California was described, and various parental factors associated with implantation before age 2 years were analyzed.

**Results:**

Among 182 children receiving cochlear implantations, the median (IQR) age was 3 (1-5) years and 58 children (31.9%) received implantations at ages 2 years or younger. There were 90 girls (49.5%) and 92 boys (50.5%), and among 170 children with race and ethnicity data, there were 27 Asian or Pacific Islander children (15.9%), 63 Hispanic children (37.1%), and 55 White children (32.4%). The risk of CI was significantly decreased among Black children compared with Asian or Pacific Islander children (relative risk [RR], 0.18 [95% CI, 0.07-0.47]; *P* = .001) and White children (RR, 0.24 [95% CI, 0.10-0.59]; *P* = .002) and among Hispanic children compared with Asian or Pacific Islander children (RR, 0.32 [95% CI, 0.21-0.50]; *P* < .001) and White children (RR, 0.42 [95% CI, 0.29-0.59; *P* < .001). Compared with private insurance, Medicaid insurance was associated with decreased odds of implantation at ages 2 years or younger (odds ratio [OR], 0.19 [95% CI, 0.06-0.64]; *P* = .007), and every 1 percentage point increase in maternal high school completion percentage in a given California hospital referral region was correlated with a 5–percentage point increase in percentage of cochlear implants performed at age 2 years or younger (*b* *=* 5.18 [95% CI, 1.34-9.02]; *P* = .008). There were no significant differences in rates of early implantation by race or ethnicity.

**Conclusions and Relevance:**

This study found significant variability in pediatric cochlear implantation rates in California. These findings suggest that socioeconomic and parental factors may be associated with differences in access to early cochlear implantation and suggest the need to invest in initiatives to address barriers to appropriate and timely access to care.

## Key Points

**Question:**

What factors are associated with delayed cochlear implantation among children in California?

**Findings:**

In this cross-sectional study of 182 children undergoing cochlear implantation, those with Medicaid insurance had decreased odds of implantation prior to age 2 years. Higher maternal education level was associated with improved early cochlear implantation rates.

**Meaning:**

These findings suggest that socioeconomic and parental factors may be associated with access to early cochlear implantation among children in California.

## Teaser

This cross-sectional study investigates the association of sociodemographic, geographic, and parental characteristics with delayed cochlear implantation among children in California.

## Introduction

According to the Center for Disease Control and Prevention 2018 Early Hearing Detection and Intervention summary data, 1.7 of every 1000 screened newborns have diagnosed hearing loss.^1^ Most such hearing loss is genetic in etiology, with autosomal recessive nonsyndromic hearing loss accounting for 80% of these hearing loss diagnoses.^2,3^ However, the prevalence of hearing loss increases into school age and adolescence, which is likely associated with progressive etiologies and environmental or acquired factors, including cytomegalovirus infection, meningitis, trauma, or ototoxicity related to chemotherapy.^3,4^

Among children with bilateral severe to profound sensorineural hearing loss (SNHL), hearing aids and amplification are not associated with sufficient benefits. In an observational study in the United Kingdom, Lovett et al^5^ found that children with bilateral cochlear implantation (CI) had increased odds of better listening outcomes compared with children with digital hearing aids when unaided pure tone means were at 80 dB hearing level or poorer. Such patients are candidates for CI and should undergo CI as soon as possible, ideally within 12 months of age.^6^ This is evidenced by numerous studies^7,8,9,10^ investigating the association of early implantation with improved language comprehension and expression, educational achievement, and satisfaction and quality of life.

However, access to early CI among children can be complicated by multiple socioeconomic and psychosocial factors. Studies^11,12,13^ using national databases have reported that race and public insurance are barriers associated with decreased rates of early pediatric CI. Moreover, maternal education and parenting have been identified as potential factors associated with rates of early CI, as well as developmental and language outcomes after implantation.^9,14^

The purpose of this study was 3-fold: to describe the epidemiology of pediatric CIs in California from individual and geographic frameworks, to identify sociodemographic factors associated with early CI, and to investigate the association of maternal education and parenting with early CI. While prior analyses have described demographics of children receiving CIs, this study is the first, to our knowledge, to describe geographic variation and investigate the association of maternal factors with early CI at a statewide level.

## Methods

This study was approved by the University of California, Los Angeles, Institutional Review Board, which waived the requirement for informed consent, assent, and parental permission under 45 CFR §46.116^15^ for this research. The study adheres to the Strengthening the Reporting of Observational Studies in Epidemiology (STROBE) reporting guideline for cross-sectional studies.

### Study Population

The Healthcare Cost and Utilization Project (HCUP) State Ambulatory Surgery and Services Databases (SASD) are state-specific databases that include all ambulatory surgical procedures and other outpatient services performed at hospital-owned facilities in a given calendar year. Each database contains publicly available, deidentified, encounter-level discharge information, including demographics, payer status, *International Statistical Classification of Diseases and Related Health Problems, Tenth Revision *(*ICD-10*) diagnosis codes, and *Current Procedural Terminology *(*CPT*) codes for each encounter.

The California SASD for calendar year 2018 was obtained. All patients who underwent CI (*CPT* 69930) and were aged 9 years or younger were identified and included in the final cohort. Descriptive characteristics obtained from the HCUP SASD included age, race, sex, zip code of residence, primary payer status, median household income based on zip code, and metropolitan county status. Per HCUP SASD documentation, the variables of race and ethnicity and sex are provided by the data source or participant and are recoded into a uniform coding scheme. Information on race and ethnicity came from the database, which collected race and ethnicity data from self-report. Race and ethnicity were assessed because they have been associated with differences in CI rates in prior studies. Information regarding medical centers at which procedures were performed is not available in the California SASD. In accordance with the HCUP data use agreement, all categories with 10 or fewer individuals were masked.

### Incidence Rates in Cochlear Implantation by Race and Insurance

We first investigated CI rates by race, as done in prior studies.^13,16^ The 2018 American Community Survey data through the US Census Bureau was used to measure the total number of children aged 9 years or younger in California.^17^ Based on prior studies, we then assumed that 0.04% of these children had severe to profound SNHL.^2,18^ Finally, we estimated the number of children with severe to profound SNHL by race and ethnicity using estimates from the Gallaudet Research Institute demographic study data. This annual survey collects national data from public and private schools and programs that provide services for children who are deaf or hard of hearing and contains information for approximately 40 000 children; we used the 2009 to 2010 summary data report because it was the most recent report to provide state-specific racial and ethnic breakdowns.^19^

For each race or ethnicity, we calculated the incidence of CIs per 1000 children with severe to profound SNHL. The relative risk of CI among different races and ethnicities was measured. We performed a similar calculation to measure the CI incidence rate and compare between private and Medicaid insurance.

### Statistical Analysis

#### Factors Associated With Early CI

Next, we sought to identify sociodemographic variables associated with earlier implantation. To conduct this analysis, we generated a binary outcome variable measuring whether CI was performed at age 2 years or younger. While the general consensus now advocates for CI prior to age 12 months among children with bilateral severe to profound SNHL,^6^ the Food and Drug Administration (FDA) did not approve decreasing the age from 12 months to 9 months until 2019. Evaluation of CI at age younger than 1 year thus could not be done owing to inadequate sample size. A binary logistic regression analysis was conducted to determine socioeconomic factors that were independently associated with CI at ages 2 years or younger. Age, race, sex, insurance, income quartile, and urban or rural index as defined in the data set were included as covariates in the multivariable model.

#### Geographic Trends Among Hospital Referral Regions

All patients were aggregated into hospital referral regions (HRRs) to describe geographic practice patterns. These regions are geographic delineations created by the Dartmouth Atlas of Health Care to define unique health care markets; they have been used across specialties to characterize geographic variations in medical practice patterns. A zip-to-HRR crosswalk was used to link zip codes to the appropriate HRRs.^20^

We measured 2 outcomes for each HRR: the incidence of CIs per 1000 children with severe to profound SNHL and the percentage of pediatric CIs performed when the children were ages 2 years or younger. The first outcome was measured using similar methods as those previously described. The number of children aged 9 years or younger for each HRR was measured using the 2018 American Community Survey, and the estimated number of children with severe to profound SNHL for each HRR was calculated using the same 0.04% estimate as described previously. This information was used to measure the incidence of CIs per 1000 children with severe to profound SNHL for each HRR.

Finally, among HRRs, we analyzed the association of general education levels, maternal education levels, and percentage of single mother households with age at CI. These data were obtained from the 2018 American Community Survey. A multivariable generalized linear model with logit link and binomial family was generated to account for the nonlinear nature of the dependent variable and investigate whether differences in these variables were associated with the percentage of CIs performed among children aged 2 years or younger in a given HRR. Covariates included rates of general workforce high school completion, maternal high school completion, and number of unmarried, unpartnered mothers in the previous 12 months.

#### Sensitivity Analysis

To better include patients with congenital and early onset hearing loss who would be candidates for early implantation (and exclude those with likely delayed or progressive hearing loss), we performed a sensitivity analysis including only children who received implantations at ages 5 years or younger. A binary logistic regression as described previously was repeated for this subgroup. In addition, using age as a continuous variable in this subgroup, we conducted a multivariable linear regression to quantify the difference in age of implantation by these demographic variables.

All statistical analysis and data visualization were performed using Stata statistical software version 14.0 (StataCorp) and ArcGIS software version 10.8.1 (Esri). All statistical tests were 2-sided, and an α level of significance of .05 was set a priori for such analyses detailed previously. Data were analyzed from March through August 2021.

## Results

### Patient Characteristics

**Figure 1.**
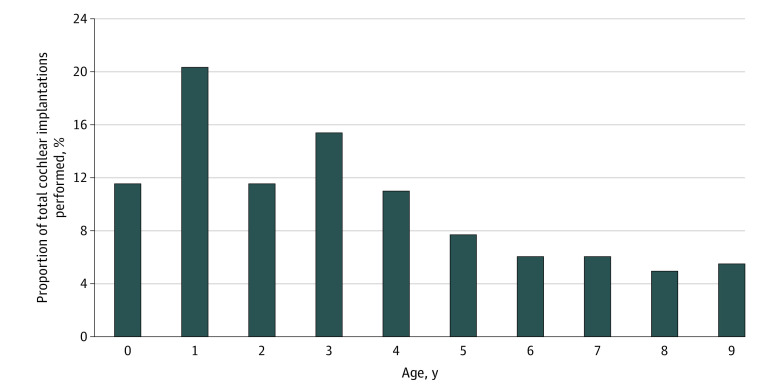
Distribution of Age at Cochlear Implantation in California in 2018

Among 182 children who underwent CIs in California in 2018, the median (IQR) age of implantation was 3 (1-5) years and the mean (SD) age of implantation was 3.4 (2.6) years. Implantations were performed among 21 patients (11.5%) at ages 1 year or younger and 58 patients (31.9%) at ages 2 years or younger. The distribution of ages at which CI was performed is detailed in Figure 1. There were 90 girls (49.5%) and 92 boys (50.5%), and among 170 children with race and ethnicity data, there were 27 Asian or Pacific Islander children (15.9%), 63 Hispanic children (37.1%), 55 White children (32.4%), and 20 children with other race or ethnicity (11.8%). Racial and ethnic groups with 10 or fewer patients were masked for patient confidentiality. Demographics of the patient population are presented in Table 1.

**Table 1.** Patient Characteristics

Characteristic	Patients, No. (%) (N = 182)^a^
Age, y	
≤2	58 (31.9)
>2	124 (68.1)
Race and ethnicity	
No. with data	170
Asian or Pacific Islander	27 (15.9)
Black	NA
Hispanic	63 (37.1)
White	55 (32.4)
Other^b^	20 (11.8)
Sex	
Girls	90 (49.5)
Boys	92 (50.5)
Income, quartile	
No. with data	181
First	45 (24.9)
Second	44 (24.3)
Third	35 (19.3)
Fourth	57 (31.5)
Insurance	
Medicaid	62 (34.1)
Private	94 (51.6)
Other	26 (14.3)
Urban or rural status	
Metropolitan county, >1 million population	
Central	112 (61.5)
Fringe	24 (13.2)
Metropolitan county, <1 million population	44 (24.2)
Nonmetropolitan	NA

Abbreviation: NA, not applicable.

^a^ All cells with 10 or fewer individuals were masked for patient confidentiality.

^b^ Other was defined by the variable race in the original Healthcare Cost and Utilization Project State Ambulatory Surgery and Services Databases data set. Groups within the category other were not defined per the data set’s description of the variable race/other.

### Incidence Rates in Cochlear Implantation by Race or Ethnicity and Insurance

To analyze differences in CI rates in California in 2018 by race and ethnicity, the incidence rate of CIs per 1000 children with SNHL was calculated for each racial or ethnic group. The highest incidence rates were among Asian or Pacific Islander children (87.4 CIs per 1000 children) and White children (67.5 CIs per 1000 children), compared with incidence rates among Hispanic and Black children of 28.1 CIs and 16.0 CIs per 1000 children, respectively. The relative risk (RR) of receiving a CI by race and ethnicity is presented in Table 2, with White and Asian or Pacific Islander children as reference groups. While there were no significant differences between White and Asian or Pacific Islander children, the risk of CI was significantly decreased among Black children compared with Asian or Pacific Islander children (RR, 0.18 [95% CI, 0.07-0.47]; *P* = .001) and White children (RR, 0.24 [95% CI, 0.10-0.59]; *P* = .002) and among Hispanic children compared with Asian or Pacific Islander children (RR, 0.32 [95% CI, 0.21-0.50]; *P* < .001) and White children (RR, 0.42 [95% CI, 0.29-0.59; *P* < .001).

**Table 2.** Relative Rates of Pediatric Cochlear Implantation by Race and Ethnicity

Race or ethnicity	RR (95% CI)	*P* value
Asian or Pacific Islander as reference		
Black	0.18 (0.07-0.47)	.001
Hispanic	0.32 (0.21-0.50)	<.001
White	0.77 (0.50-1.20)	.25
White as reference		
Asian or Pacific Islander	1.29 (0.83-2.01)	.25
Black	0.24 (0.10-0.59)	.002
Hispanic	0.42 (0.29-0.59)	<.001

Abbreviation: RR, relative risk.

There was no significant difference in incidence rates of CIs stratified by insurance. The incidence rate among individuals with private insurance was 43.0 CIs per 1000 children, compared with 37.8 CIs per 1000 children for individuals with Medicaid insurance (RR, 0.88 [95%, CI 0.64-1.20]; *P* = .42).

### Factors Associated With Early CI

A binary logistic regression analysis was performed to compare children undergoing implantation at ages 2 years or younger vs at age 2 years (Table 3). Individuals with Medicaid insurance had decreased odds of implantation at age 2 years or younger (odds ratio [OR], 0.19 [95% CI, 0.06-0.64]; *P* = .007) compared with individuals with private insurance. No significant differences by race or ethnicity in odds of CI at ages 2 years or younger were observed, in contrast to the differences observed in overall CI incidence rates.

**Table 3.** Binary Logistic Regression of Factors Associated With Early Cochlear Implantation

Factor	OR (95% CI)	*P* value
Race and ethnicity		
White	1 [Reference]	NA
Asian or Pacific Islander	0.86 (0.29-2.56)	.78
Black	0.95 (0.07-13.1)	.97
Hispanic	1.58 (0.54-4.63)	.4
Other^a^	1.67 (0.54-5.23)	.37
Female sex	1.72 (0.81-3.62)	.16
Insurance		
Private	1 [Reference]	NA
Medicaid	0.19 (0.06-0.64)	.007
Other	0.49 (0.16-1.54)	.22
Median income, quartile		
First	1 [Reference]	NA
Second	2.31 (0.58-9.10)	.23
Third	2.48 (0.59-10.5)	.22
Fourth	2.24 (0.50-10.1)	.29
Urban or rural status		
Metropolitan county, >1 million population		
Central	1 [Reference]	NA
Fringe	0.77 (0.28-2.22)	.63
Metropolitan county, <1 million population	0.75 (0.27-2.10)	.58
Nonmetropolitan	12.7 (0.58-280)	.11

Abbreviations: NA, not applicable; OR, odds ratio.

^a^ Other was defined by the variable race in the original Healthcare Cost and Utilization Project State Ambulatory Surgery and Services Databases data set. Groups within the category other were not defined per the data set’s description of the variable race/other.

This finding was verified on sensitivity analysis examining only children aged 5 years or younger (eTables 1 and 2 in the Supplement). Children with Medicaid had nearly one-fifth the odds of undergoing implantation prior to age 2 years (OR, 0.19 [95% CI 0.05-0.68]; *P* = .01). In linear regression, Medicaid insurance was associated with implantation nearly 1 year later in life compared with private insurance (*b =* 0.93 [95% CI, 0.09-1.77]; *P* = .03).

### Geographic Trends Among HRRs

**Figure 2.**
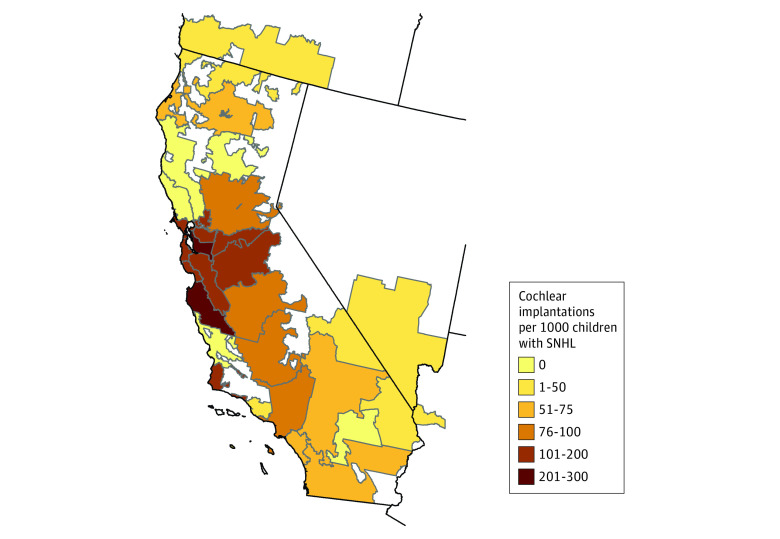
Geographic Density Map of Cochlear Implantation Incidence Rates in California The number of cochlear implantations per 1000 children aged younger than 9 years with severe to profound sensorineural hearing loss among California hospital referral regions is presented.

An analysis of geographic patterns of CIs was conducted (Figure 2). The maximum number of CIs in a California HRR was 289.1 CIs per 1000 children with severe to profound SNHL. The median (IQR) number of CIs per HRR was 104.4 (69.4-161.6) CIs per 1000 children.

Rates of early pediatric CIs were then evaluated by general workforce education, maternal education, and single mother status among HRRs. While there were no correlations between general workforce education (*b* = −3.23 [95% CI, −6.88 to 0.42]; *P* = .08) or maternal marriage status (*b* = 0.70 [95% CI, −0.98 to 2.37]; *P* = .42) and CI rates, every 1 percentage point increase in maternal high school completion percentage in a HRR was correlated with a 5 percentage point increase in the percentage of CIs performed at ages 2 years or younger (*b =* 5.18 [95% CI, 1.34-9.02]; *P* = .008).

## Discussion

In this cross-sectional study investigating demographics among children undergoing CI and detailed geographic variation in rates of CI across HRRs in California in 2018, Medicaid insurance was associated with decreased odds of CI at ages 2 years or younger. Additionally, we investigated the association of maternal education level with CI rates and found that geographic areas with increased maternal education levels were associated with increased rates of early implantation. To our knowledge, our study is the first to investigate this association at the statewide level.

The association of lower socioeconomic status with decreased rates of pediatric CI has been well established. Armstrong et al^21^ performed a retrospective review of 57 children receiving CI and found that public insurance was associated with delayed SNHL diagnosis and delayed time from diagnosis to implantation. Using the Kids’ Inpatient Database, Stern et al^16^ and Tampio et al^13^ found that White and Asian or Pacific Islander children received implantations at significantly increased rates compared with Hispanic and Black children and that children receiving implantations were more likely to live in zip codes with higher median incomes. A study^11^ of 1511 pediatric CIs in Florida from 2005 to 2017 reported that Black and Hispanic children and those with Medicaid were less likely to receive implantations before age 2 years. However, 454 implantations were excluded from the study because of missing demographics for the children, and it is unclear whether these were from earlier years or all years. Additionally, the authors did not include Asian or Pacific Islander individuals in their cohort. Of note, the FDA only recently approved CIs among children aged younger than 12 months, doing so in 2019; all implantations occurring among children at younger than age 1 year in this outcome variable were thus off-label at the time they were performed.

Our analysis found that in California, Asian or Pacific Islander and White children received implantations at more than 2-fold the rate among Black or Hispanic children, which is consistent with racial disparities reported by Tampio et al.^13^ However, there were no significant differences in likelihood of early implantation by race or ethnicity. Furthermore, in direct comparison between the present study using a 2018 California database and those of Tampio et al^13^ and Stern et al,^16^ which used older databases, there was increased incidence of pediatric CIs in every racial and ethnic group. This may be associated with the use of different data sets, but it may also be partially associated with improvements in access to CIs in the last 10 to 20 years and suggest a step toward more equitable access to care.

We found that while the overall incidence rate was similar among children with private vs Medicaid insurance, children with Medicaid insurance were less likely to have undergone CIs before age 2 years. This suggests that access to CIs may still be delayed in Medicaid populations, which may be associated with profound differences in childhood language development. Additionally, there were no associations of race or ethnicity, sex, or urban vs rural status with early CI. There were decreased rates of early CIs among patients in the poorest income quartile, but this difference was not statistically significant. Owing to sample size and lack of availability of multiple years of the California SASD, we were unable to study temporal trends or interaction variables. However, more equitable access over the last decade has been noted in studies analyzing temporal patterns.^11,13^ This suggests that continued efforts are needed to identify children who are candidates for CI as early as possible and address barriers from the moment of newborn hearing screenings toward appropriate evaluation and ultimate implantation, particularly in these high-risk populations.

Our study provides a unique perspective on geographic variations within California. We identified significant variability among HRRs, and while the sociodemographic factors described previously may certainly be associated with these outcomes, a multitude of unmeasured variables associated with rates of pediatric CIs exists. One such factor may be maternal education. There are already numerous studies detailing the association of maternal education with improved language outcomes among children after CI. Marnane and Ching in 2015^22^ analyzed a cohort of 116 children who received CIs at ages 3 years or younger in Australia, finding that a higher maternal education level was associated with increased usage of the device and improved linguistic outcomes. Szagun and Stumper^23^ studied the linguistic progress of a cohort of 25 children, with follow-up done as late as 30 months after implantation, and similarly found that higher levels of maternal education were associated with more rapid linguistic progress, regardless of age at implantation.

We found that HRRs with increased maternal education levels were associated with increased rates of CI among children prior to age 2 years. To our knowledge, no prior studies outside of small cohort analyses have found this association. Maternal education may be associated not only with language outcomes postprocedure, but also with the likelihood of a child receiving an implant at an early age. In a detailed medical record analysis by Armstrong et al,^21^ the most common barriers to early implantation were parental factors, including delayed or missed appointments, difficulty navigating the health care system, misunderstanding of the candidacy process, and reluctance to have their children undergo surgical treatment. Several of these reasons may, in part, be associated with parental education level, and identification of these at-risk patient populations by pediatricians, audiologists, and otolaryngologists is necessary to provide targeted efforts and increased awareness among parents regarding the importance of timely implantation. Unfortunately, we cannot delve further into the factors associated with this finding given the data set’s nature; this outcome may be associated with decreased access to services that appropriately evaluate such children, in conjunction with increased time with hearing aids and decreased awareness of CI technology. While this is a cross-sectional analysis, given the critical association of age of implantation with language development and future achievement, this finding further suggests the association of socioeconomic factors with future success among children with profound SNHL.

### Limitations

Our study has several limitations. First, the HCUP SASD is an administrative database and is subject to coding errors; this is likely associated with a small difference in outcome given the specific inclusion criteria used in our study. Second, our study was cross-sectional and could not evaluate causality, including in associations between maternal education levels and age at CI. Third, there may be factors unique to California which make our findings less generalizable to the wider, national population, such as differences in racial and ethnic demographics or governmental policy (eg, Medicaid eligibility); however, our findings are aligned with those of prior studies cited. Fourth, unlike the previously cited study by Liu et al,^11^ the only available calendar year for the California SASD was 2018, and we could not evaluate temporal trends. Fifth, we lacked information regarding workup and prior treatments of children who underwent implantation; there were no data regarding newborn hearing screening results, age at which hearing aids were used, progression of hearing loss, or time course of initial audiologic evaluation by an otolaryngologist. We also lacked information regarding clinical decision-making. These factors are crucial toward better understanding barriers to children with severe to profound hearing loss undergoing implantation at an appropriate age. Additionally, drawing extensive conclusions using a population-based cross-sectional study is subject to ecological fallacy and should be made with prudence and acknowledgment of this limitation.

## Conclusions

We have described the geographic variation of pediatric CI and have presented evidence suggesting the association between insurance and early implantation. We have found that maternal education was associated with timely treatment for children with hearing loss. Findings concerning these inequalities further suggest the complicated socioeconomic barriers that exist for these patients and their families and the need to invest in multidisciplinary teams and educational, research, and governmental policy initiatives.

## Notes

Supplement. **eTable 1.** Binary Logistic Regression of Factors Associated With Cochlear Implantation at Ages 2 Years or Younger Among Children Ages 5 Years or Younger **eTable 2.** Multivariate Linear Regression of Factors Associated With Age at Cochlear Implantation Among Children Ages 5 Years or Younger

## References

[1] Centers for Disease Control and Prevention. Data and statistics about hearing loss in children. Accessed April 6, 2021. https://www.cdc.gov/ncbddd/hearingloss/data.html

[2] Marazita ML, Ploughman LM, Rawlings B, Remington E, Arnos KS, Nance WE. Genetic epidemiological studies of early-onset deafness in the U.S. school-age population. Am J Med Genet. 1993;46(5):486-491. doi:10.1002/ajmg.1320460504 8322805

[3] Korver AM, Smith RJ, Van Camp G, . Congenital hearing loss. Nat Rev Dis Primers. 2017;3:16094. doi:10.1038/nrdp.2016.94 28079113 PMC5675031

[4] Koomen I, Grobbee DE, Roord JJ, Donders R, Jennekens-Schinkel A, van Furth AM. Hearing loss at school age in survivors of bacterial meningitis: assessment, incidence, and prediction. Pediatrics. 2003;112(5):1049-1053. doi:10.1542/peds.112.5.1049 14595044

[5] Lovett RE, Vickers DA, Summerfield AQ. Bilateral cochlear implantation for hearing-impaired children: criterion of candidacy derived from an observational study. Ear Hear. 2015;36(1):14-23. doi:10.1097/AUD.0000000000000087 25170781

[6] American Academy of Otolaryngology-Head and Neck Surgery. Position statement: pediatric cochlear implantation candidacy. Accessed April 6, 2021. https://www.entnet.org/resource/position-statement-pediatric-cochlear-implantation-candidacy/

[7] Ganek HV, Feness ML, Goulding G, . A survey of pediatric cochlear implant recipients as young adults. Int J Pediatr Otorhinolaryngol. 2020;132:109902. doi:10.1016/j.ijporl.2020.109902 32006862

[8] Yoshinaga-Itano C, Sedey AL, Wiggin M, Mason CA. Language outcomes improved through early hearing detection and earlier cochlear implantation. Otol Neurotol. 2018;39(10):1256-1263. doi:10.1097/MAO.0000000000001976 30444842

[9] Spencer LJ, Tomblin JB, Gantz BJ. Growing up with a cochlear implant: education, vocation, and affiliation. J Deaf Stud Deaf Educ. 2012;17(4):483-498. doi:10.1093/deafed/ens024 22949609 PMC3459294

[10] Venail F, Vieu A, Artieres F, Mondain M, Uziel A. Educational and employment achievements in prelingually deaf children who receive cochlear implants. Arch Otolaryngol Head Neck Surg. 2010;136(4):366-372. doi:10.1001/archoto.2010.31 20403853

[11] Liu X, Rosa-Lugo LI, Cosby JL, Pritchett CV. Racial and insurance inequalities in access to early pediatric cochlear implantation. Otolaryngol Head Neck Surg. 2021;164(3):667-674. doi:10.1177/0194599820953381 32930656

[12] Huang Z, Gordish-Dressman H, Preciado D, Reilly BK. Pediatric cochlear implantation: variation in income, race, payer, and charges across five states. Laryngoscope. 2018;128(4):954-958. doi:10.1002/lary.26686 28599062

[13] Tampio AJF, Schroeder Ii RJ, Wang D, Boyle J, Nicholas BD. Trends in sociodemographic disparities of pediatric cochlear implantation over a 15-year period. Int J Pediatr Otorhinolaryngol. 2018;115:165-170. doi:10.1016/j.ijporl.2018.10.003 30368379

[14] Quittner AL, Cruz I, Barker DH, Tobey E, Eisenberg LS, Niparko JK; Childhood Development after Cochlear Implantation Investigative Team. Effects of maternal sensitivity and cognitive and linguistic stimulation on cochlear implant users’ language development over four years. J Pediatr. 2013;162(2):343-8.e3. doi:10.1016/j.jpeds.2012.08.003 22985723 PMC3638743

[15] General requirements for informed consent. 45 CFR §46.116 (2018). Accessed December 6, 2021. https://www.ecfr.gov/current/title-45/subtitle-A/subchapter-A/part-46/subpart-A/section-46.116

[16] Stern RE, Yueh B, Lewis C, Norton S, Sie KC. Recent epidemiology of pediatric cochlear implantation in the United States: disparity among children of different ethnicity and socioeconomic status. Laryngoscope. 2005;115(1):125-131. doi:10.1097/01.mlg.0000150698.61624.3c 15630380

[17] US Census Bureau. American Community Survey (ACS). Accessed April 1, 2021. https://www.census.gov/programs-surveys/acs

[18] Blanchfield BB, Feldman JJ, Dunbar JL, Gardner EN. The severely to profoundly hearing-impaired population in the United States: prevalence estimates and demographics. J Am Acad Audiol. 2001;12(4):183-189. 11332518

[19] Gallaudet Research Institute. 2009-10 State summary. Accessed December 2, 2021. https://research.gallaudet.edu/Demographics/States/2010/MA.pdf

[20] Dartmouth Atlas Project. Supplemental data. Dartmouth Atlas Data. Accessed April 6, 2021. https://atlasdata.dartmouth.edu/downloads/supplemental#crosswalks

[21] Armstrong M, Maresh A, Buxton C, . Barriers to early pediatric cochlear implantation. Int J Pediatr Otorhinolaryngol. 2013;77(11):1869-1872. doi:10.1016/j.ijporl.2013.08.031 24035734

[22] Marnane V, Ching TY. Hearing aid and cochlear implant use in children with hearing loss at three years of age: predictors of use and predictors of changes in use. Int J Audiol. 2015;54(8):544-551. doi:10.3109/14992027.2015.1017660 25816866 PMC4527863

[23] Szagun G, Stumper B. Age or experience: the influence of age at implantation and social and linguistic environment on language development in children with cochlear implants. J Speech Lang Hear Res. 2012;55(6):1640-1654. doi:10.1044/1092-4388(2012/11-0119) 22490622

