# Supplementary material for: Association of Socioeconomic Characteristics With Receipt of Pediatric Cochlear Implantations in California
Source: JAMA Netw Open. 2022 Jan 14;5(1):e2143132. doi: 10.1001/jamanetworkopen.2021.43132 (PMC8760613; doi:10.1001/jamanetworkopen.2021.43132)
Supplement: Supplement. — eTable 1. Binary Logistic Regression of Factors Associated With Cochlear Implantation at Ages 2 Years or Younger Among Children Ages 5 Years or Younger eTable 2. Multivariate Linear Regression of Factors Associated With Age at Cochlear Implantation Among Children Ages 5 Years or Younger [file jamanetwopen-e2143132-s001.pdf]

Supplemental Online Content

Fujiwara RJT, Ishiyama G, Ishiyama A. Association of socioeconomic characteristics with receipt of pediatric cochlear implantations in California. *JAMA Netw Open*. 2022;5(1):e2143132. doi:10.1001/jamanetworkopen.2021.43132

eTable 1. Binary Logistic Regression of Factors Associated With Cochlear Implantation at Ages 2 Years or Younger Among Children Ages 5 Years or Younger
eTable 2. Multivariate Linear Regression of Factors Associated With Age at Cochlear Implantation Among Children Ages 5 Years or Younger

This supplemental material has been provided by the authors to give readers additional information about their work.

© 2022 Fujiwara RJT et al. *JAMA Network Open.*

eTable 1. Binary Logistic Regression of Factors Associated With Cochlear Implantation at Ages 2 Years or Younger Among Children Ages 5 Years or Younger

	Odds Ratio (95% CI)	p value
Race		
White	1	
Hispanic	1.70 (0.55-5.23)	0.35
Asian	1.20 (0.36-3.95)	0.76
Black	0.77 (0.05-11.16)	0.85
Other	2.03 (0.57-7.23)	0.27
Sex: Female	1.25 (0.55-2.84)	0.59
Insurance		
Private	1	
Medicaid	0.19 (0.05-0.68)	0.01
Other	0.43 (0.13-1.48)	0.18
Median income quartile		
First	1	
Second	2.02 (0.46-8.93)	0.07
Third	2.11 (0.46-9.69)	0.34
Fourth	1.43 (0.29-6.97)	0.66
Urban/Rural Status		
Central metropolitan county, >1 million population	1	
Fringe metropolitan county, >1 million population	0.66 (0.22-2.01)	0.47
Metropolitan county, <1 million population	0.75 (0.25-2.31)	0.62
Non-metropolitan	7.01 (0.31-160.7)	0.22

© 2022 Fujiwara RJT et al. *JAMA Network Open.*

eTable 2. Multivariate Linear Regression of Factors Associated With Age at Cochlear Implantation Among Children Ages 5 Years or Younger

	Coefficient (95% CI)	p value
Race		
White		
Hispanic	-0.39 (-1.16-0.38)	0.31
Asian	-0.02 (-0.91-8.75)	0.97
Black	0.10 (-1.64-1.85)	0.91
Other	-0.42 (-1.35-0.52)	0.38
Sex: Female	0.05 (-0.53-0.62)	0.88
Insurance		
Private		
Medicaid	0.93 (0.09-1.77)	0.03
Other	0.98 (0.08-1.88)	0.03
Median income quartile		
First		
Second	-0.48 (-1.43-0.47)	0.32
Third	-0.41 (-1.41-0.59)	0.42
Fourth	0.04 (-0.98-1.07)	0.93
Urban/Rural Status		
Central metropolitan county, >1 million population		
Fringe metropolitan county, >1 million population	0.49 (-0.31-1.28)	0.23
Metropolitan county, <1 million population	0.08 (-0.69-0.86)	0.83
Non-metropolitan	-1.87 (-4.22-0.48)	0.12

© 2022 Fujiwara RJT et al. *JAMA Network Open.*
